# Supplementary material for: Effects of Functional Depletion of Doublesex on Male Development in the Sawfly, Athalia rosae
Source: Insects. 2021 Sep 22;12(10):849. doi: 10.3390/insects12100849 (PMC8538284; doi:10.3390/insects12100849)
Supplement: Supplementary file 1 [file insects-12-00849-s001.zip › insects-1362065-supplementary.pdf]

insects

**Table S1.** Primer sequences and PCR conditions utilized in this study.

| Target gene | Primers | Sequence(5'→3') |
|---|---|---|
| *Ardsx* | ArdsxFMF1 | AAAGATGCACAAGCCGATTTG |
| | ArdsxFMR1 | GGCTGATGAACAAGGCTCATC |
| *Arfru* | ArfruX1F | GAGACGCAAACCAGTGCAAG |
| | ArfruX1R | TGCTGGCGATACGCATTAGA |
| | ArfruX2F | CCCAACCCTACATCACACCC |
| | ArfruX2R | TGCCTCTGCCCCTCTCATTA |
| | ArfruX3F | CCCAACCCTACATCACACCC |
| | ArfruX3R | GCTTGCACCTGTGAAGGTTG |
| | ArfruX4F | CCCAACCCTACATCACACCC |
| | ArfruX4R | CCTACATCCGATACCGGCAC |
| | ArfruX5F | GTTTGCGAAAGGGCCCATAG |
| | ArfruX5R | CTTGCACTGGTTTGCGTCTC |
| | ArfruX6F | CAACGTGTTCAGTGTCACCT |
| | ArfruX6R | CTGCGCTAAACTATGGGCCT |
| | ArfruX7F | AAGGGTTGCTTGTATCCGCA |
| | ArfruX7R | TCTCTGACTCAGGGGATGGG |
| | ArfruX8F | AGAGATTGTGGAGGTTGGGAC |
| | ArfruX8R | TTTGCAATGCTTCCGCAGTC |
| | ArfruX9F | CCCAACCCTACATCACACCC |
| | ArfruX9R | GTGCAGGACTGCTCAAGGAT |
| | ArfruX10F | TGAGCTCAAGCATTGGAGGA |
| | ArfruX10R | TGTTAAGCTGAGCGCTACGA |
| *EF-1* | ArEF1-LP | CTTCACTCTTGGTGTCAAGCAGCTC |
| | ArEF1-RP | ACATCCTGAAGAGGAAGACGGAGAG |

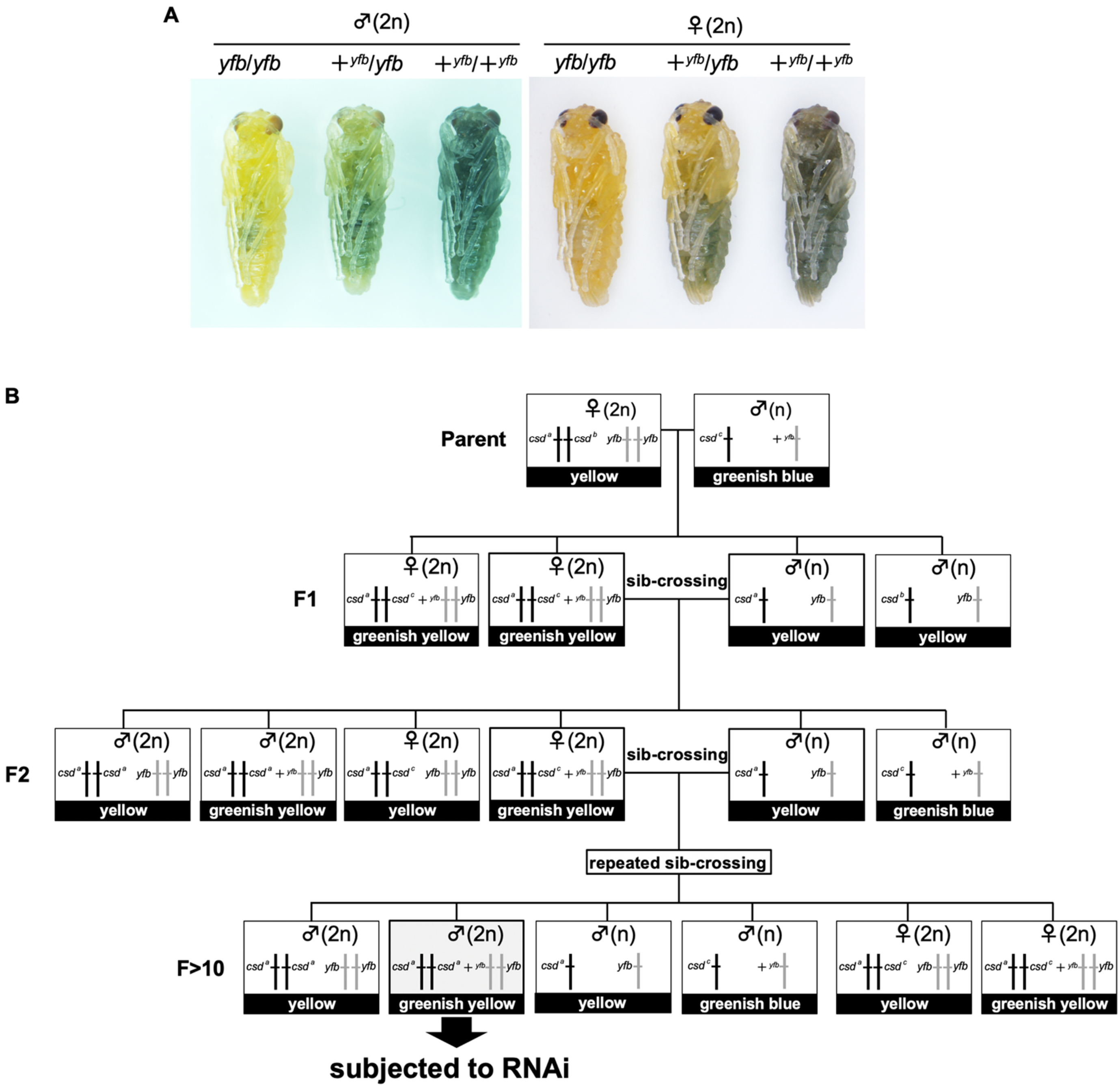


**Figure S1.** Crossing scheme to obtain diploid males. (A) Female and diploid male pupae with the indicated genotype. The apparent color of the pupae immediately after pupation reflects the color of their fat bodies due to the transparency of the epidermis. Wild-type pupae ($+^{yfb}/+^{yfb}$) are greenish blue, whereas pupae homozygous for the *yfb* mutation (*yfb*/*yfb*) are yellow. Pupae heterozygous for the *yfb* mutation ($+^{yfb}$/ *yfb*) display intermediate coloration (greenish yellow) [62]. (B) Crossing scheme to obtain diploid males used for complete RNAi. A single diploid female homozygous for *yfb* (*yfb*/*yfb*) was mated with a wild-type haploid male, and their progeny was subjected to sib-crossing, in which a single diploid female ($+^{yfb}$/*yfb*) was crossed with a single haploid male ($+^{yfb}$ or *yfb*). Such interbreeding was repeated several times. The resulting males heterozygous for *yfb* ($+^{yfb}$/*yfb*) were treated as diploid males and subjected to complete RNAi treatment.

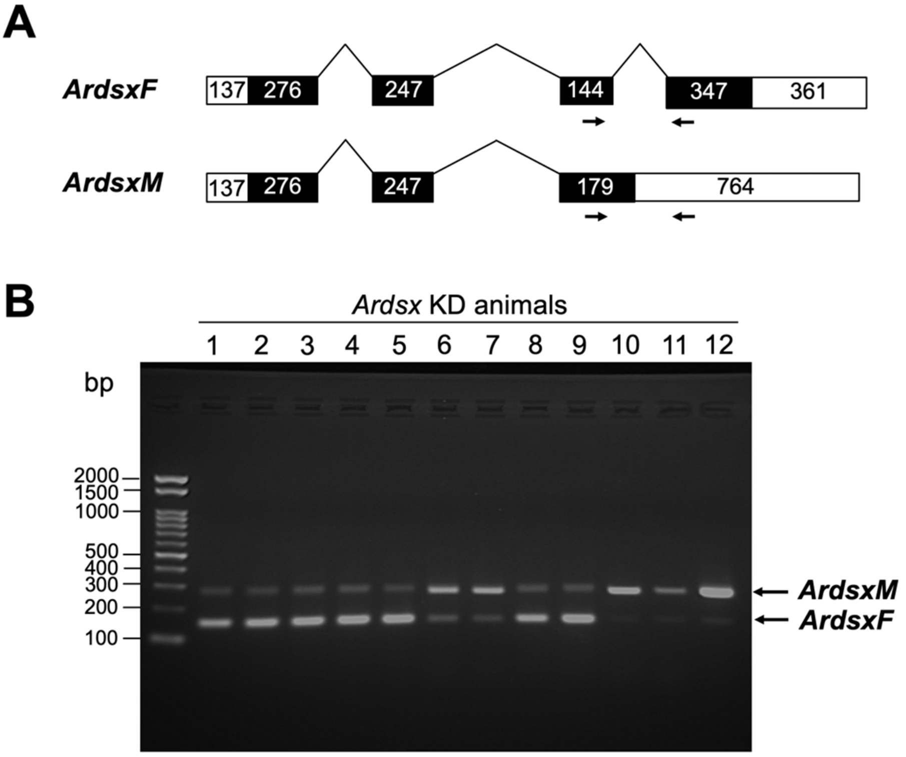


| sex | individual number |
|---|---|
| female | 1, 2, 3, 4, 5, 8, 9 |
| male | 6, 7, 10, 11, 12 |

**Figure S2.** Molecular sexing of *Ardsx*-knockdown diploid animals. (A) Schematic diagram of sex-specific splice variants of *Ardsx*. Boxes represent exons. White regions indicate UTRs. Black regions represent ORFs. Arrows indicate the approximate position of the primers used for the RT-PCR described below. In the female isoform, the male-specific 119-bp sequence, which contains a stop codon, was spliced out, causing a difference in the amino-acid sequence in the C-terminal region between the male and female isoforms. (B) Sexing of each animal was determined according to the expression pattern of *Ardsx* as investigated using RT-PCR more than 7 days after adult emergence, when the effect of *Ardsx* knockdown had disappeared. The photograph shows results of RT-PCR with representative animals. Individuals that expressed female-type *Ardsx* were identified as females, whereas animals that expressed the male-specific isoform of *Ardsx* were identified as males. The amplified products were separated using 2% agarose gel electrophoresis and visualized with 1% ethidium bromide.

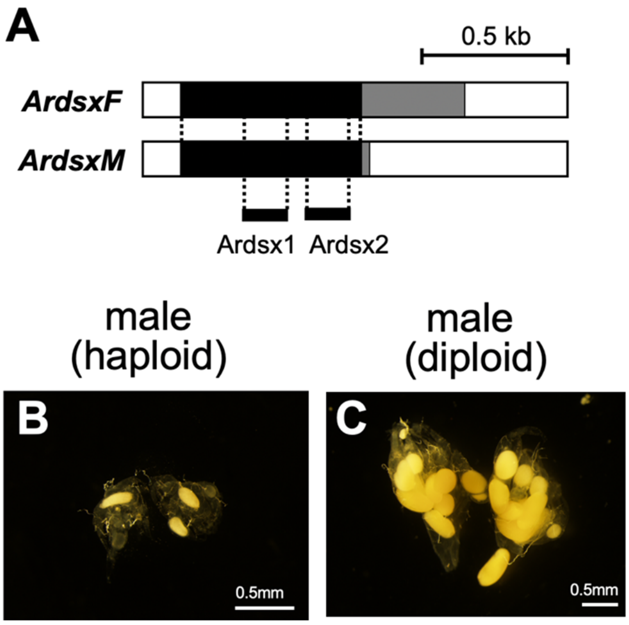


**Figure S3.** Effects of repeated injections of Ardsx2 dsRNA on sexual development of internal genital organs. (A) Schematic diagram of the *ArdsxF* and *ArdsxM* mRNAs. The white regions indicate untranslated regions (UTRs). The black regions represent the open-reading frames (ORFs) shared between the *ArdsxF* and *ArdsM* mRNAs. The gray regions indicate mRNA sequence-encoding sex-specific ORFs. Positions of the dsRNAs targeting *Ardsx* used in this study (Ardsx1 and Ardsx2) are indicated by bold lines. (B, C) Ventral view of the internal genitalia at the adult stage. *Ardsx*-knockdown haploid male (B), and diploid male (C) obtained via repeated injections of Ardsx2 dsRNA.
